# Compensatory Recovery after Multisensory Stimulation in Hemianopic Patients: Behavioral and Neurophysiological Components

**DOI:** 10.3389/fnsys.2016.00045

**Published:** 2016-05-24

**Authors:** Paolo A. Grasso, Elisabetta Làdavas, Caterina Bertini

**Affiliations:** ^1^Department of Psychology, University of BolognaBologna, Italy; ^2^Centro Studi e Ricerche in Neuroscienze Cognitive (CsrNC), Centre for Studies and Research in Cognitive Neuroscience, University of BolognaCesena, Italy

**Keywords:** hemianopia, multisensory integration, P3, visual rehabilitation, superior colliculus

## Abstract

Lateralized post-chiasmatic lesions of the primary visual pathway result in loss of visual perception in the field retinotopically corresponding to the damaged cortical area. However, patients with visual field defects have shown enhanced detection and localization of multisensory audio-visual pairs presented in the blind field. This preserved multisensory integrative ability (i.e., crossmodal blindsight) seems to be subserved by the spared retino-colliculo-dorsal pathway. According to this view, audio-visual integrative mechanisms could be used to increase the functionality of the spared circuit and, as a consequence, might represent an important tool for the rehabilitation of visual field defects. The present study tested this hypothesis, investigating whether exposure to systematic multisensory audio-visual stimulation could induce long-lasting improvements in the visual performance of patients with visual field defects. A group of 10 patients with chronic visual field defects were exposed to audio-visual training for 4 h daily, over a period of 2 weeks. Behavioral, oculomotor and electroencephalography (EEG) measures were recorded during several visual tasks before and after audio-visual training. After audio-visual training, improvements in visual search abilities, visual detection, self-perceived disability in daily life activities and oculomotor parameters were found, suggesting the implementation of more effective visual exploration strategies. At the electrophysiological level, after training, patients showed a significant reduction of the P3 amplitude in response to stimuli presented in the intact field, reflecting a reduction in attentional resources allocated to the intact field, which might co-occur with a shift of spatial attention towards the blind field. More interestingly, both the behavioral improvements and the electrophysiological changes observed after training were found to be stable at a follow-up session (on average, 8 months after training), suggesting long-term effects of multisensory audio-visual training. These long-lasting effects seem to be subserved by the activation of the spared retino-colliculo-dorsal pathway, which promotes orienting responses towards the blind field, able to both compensate for the visual field loss and concurrently attenuate visual attention towards the intact field. These results add to previous findings the knowledge that audio-visual multisensory stimulation promote long-term plastic changes in hemianopics, resulting in stable and long-lasting ameliorations in behavioral and electrophysiological measures.

## Introduction

Visual field defects, resulting from damage to the visual structures located behind the chiasma, including primary visual cortex (V1), surrounding extrastriate cortices and optic radiations, consist of a loss of visual perception in up to one half of the visual field. Patients with visual field defects cannot see a visual stimulus presented within the blind area of the visual field. Although the ability to consciously perceive visual stimuli presented in the blind field is lost, these hemianopic patients have demonstrated the specific ability to implicitly detect or discriminate certain visual features of stimuli presented in the blind field, such as motion, color and orientation (Weiskrantz et al., 1974), as well as the emotional content of the visual signals (affective blindsight; De Gelder et al., 1999; Morris et al., 2001; Pegna et al., 2005; Bertini et al., 2013; Cecere et al., 2014). These patients can also integrate unseen visual stimuli with auditory information (crossmodal blindsight; Leo et al., 2008b). The neuronal structures and pathways sustaining implicit processing of visual signals following damage to V1 or the neural pathway feeding V1 are still under debate; this topic is very relevant for the rehabilitation of visual field defects, because the same pathways could mediate recovery of the deficit, if adequately boosted.

A wide range of evidence (Milner and Goodale, 1995) converges on the existence of two different pathways sustaining unconscious and conscious perception, and diverging at early processing stages. Specifically, one pathway—in which visual information is projected from the retina to the lateral geniculate nucleus, and then to the occipital cortex—is known to underlie conscious visual processing. On the other hand, unconscious, implicit visual processing relies on an alternative pathway, in which visual signals from the retina are projected to the superior colliculus (SC) and the pulvinar, and then to the dorsal parietal cortices. In line with this idea, diffusion tensor imaging (DTI) in humans (Tamietto et al., 2012) has demonstrated anatomical connections between the SC and the amygdala via the pulvinar. Moreover, these connections were strengthened in one patient demonstrating affective blindsight. Interestingly, this finding has also been recently confirmed in monkeys (Rafal et al., 2015). This alternative pathway, involving the SC and its dorsal-parietal projections, can also explain the crossmodal blindsight phenomenon, as the SC also has a pivotal role in mediating multisensory integrative processes (Stein and Meredith, 1993). Neurons in the SC respond mainly to the combination of multiple sensory signals when presented in spatial and temporal coincidence, and, as a result, perception is enhanced when multisensory cues are provided. Interestingly, it has been shown in hemianopic patients that unseen visual stimuli can influence perception in other sensory modalities (i.e., improving auditory localization; Leo et al., 2008b) through multisensory mechanisms. In addition, there was a significant improvement in visual detection of stimuli presented in the blind field when they were concurrently presented with spatially coincident auditory stimuli (Frassinetti et al., 2005). These findings suggest that cross-modal facilitation occurs outside conscious vision and, importantly for the present study, that it may boost the processing of visual stimuli presented in the blind field (Bolognini et al., 2005; Passamonti et al., 2009; for a review, see Làdavas, 2008). Crucially, this was demonstrated for the first time by Bolognini et al. (2005), who developed a training protocol where systematic stimulation of the visual field, over a period of training with combined audio-visual stimuli, led to long-lasting amelioration of unisensory visual orientation and detection deficits in patients with chronic post-chiasmatic lesions. In addition, the treatment was also effective at improving oculomotor parameters during visual exploration, promoting fewer fixations and re-fixations, faster and larger saccades, reduced scanpath lengths, and shorter exploration times, compared to pre-treatment performance (Passamonti et al., 2009). Notably, the training promoted a reduction in self-perceived disability in daily life activities, confirming a transfer of the effects of training to ecological environments (Bolognini et al., 2005; Passamonti et al., 2009).

Due to the multisensory nature of the treatment, the authors proposed the SC and the spared retino-colliculo-dorsal pathway as the likely neural substrates involved in the ameliorative effects. Indeed, the role of the SC both in the integration of audio-visual percepts (Stein and Meredith, 1993) and in the initiation and execution of saccades (Krauzlis et al., 2004) is well documented. Notably, electrophysiological recordings have shown that the visual responses of deep SC neurons are plastic in intact adult cats, with visually unresponsive neurons becoming responsive to visual stimuli following repeated exposure to cross-modal cues (Yu et al., 2009, 2012). Even more relevant to the plasticity of the visual system after brain damage is the finding that multisensory audio-visual stimulation enhances the effectiveness of visual inputs to the SC in cortically lesioned animals, rendering SC neurons visually responsive and once again capable of supporting visual orientation behaviors (Jiang et al., 2015). The dorsal associative cortices (AES) have been shown to be crucial for this recovery process, suggesting that audio-visual training might boost the functional cortical-midbrain circuit, strengthening the residual visual inputs from the AES to the SC, which were too weak to drive SC neuron responses before training (Jiang et al., 2015).

From a rehabilitative perspective, it is also important to stress that, in association with perceptual deficits, hemianopic patients also exhibit an attentional bias towards the ipsilesional visual hemifield (Mattingley et al., 1994; Tant et al., 2002). This phenomenon seems to result from the disruption of interhemispheric fibers that keep competition between the two hemispheres in a state of equilibrium (Sprague, 1966; Kinsbourne, 2003; Cazzoli et al., 2009). In this light, hemianopics’ visual performance could be worsened by the concurrent attentional bias towards the ipsilesional visual field (Poggel et al., 2006), making it difficult to implement compensatory ocular strategies for exploring the blind visual field.

A recent study (Dundon et al., 2015) has shown that exposure to the audio-visual rehabilitative protocol used in previous studies (Bolognini et al., 2005; Làdavas, 2008; Passamonti et al., 2009) improves visual scanning behavior towards the blind field, and concurrently reduces the attentional bias towards the ipsilesional visual field. Indeed, post-training improvements in visual performance in the blind visual field co-occurred with a reduction of the P3 amplitude in response to stimuli presented in the intact field. Given that the P3 amplitude reflects the amount of attention allocated to stimulus processing (Isreal et al., 1980; Johnson, 1984, 1986), these results suggest a critical role for multisensory audio-visual treatment in reducing attentional processing of stimuli presented in the intact field.

Thus, the aim of the present study is to assess whether pairing gaze-evoking auditory cues with undetectable visual cues in a perimetry device reinstates long-lasting basic visual and visuomotor competencies in hemianopic patients, and whether this amelioration is accompanied by long-term modulation of visual spatial attention. Replicating the post-treatment results from behavioral (Bolognini et al., 2005; Passamonti et al., 2009) and electrophysiological measures (Dundon et al., 2015) at a follow-up session would confirm that a complete course of multisensory stimulation in the blind visual field is able not only to reinstate long-term compensatory saccadic eye movements towards the blind field, but also to induce long-term modulation of visuospatial attention allocation; these findings would indicate long-term plastic changes in the neural structures involved in recovery. Patients underwent a course of multisensory treatment for 2 weeks, and their behavioral performance and electrophysiological measures were tested at four time points: baseline 1 (before training), baseline 2 (2 weeks after baseline 1, and immediately before training to control for possible practice effects), post (immediately after training) and follow-up (8 months after training, on average).

## Materials and Methods

### Participants

**Figure 1:**
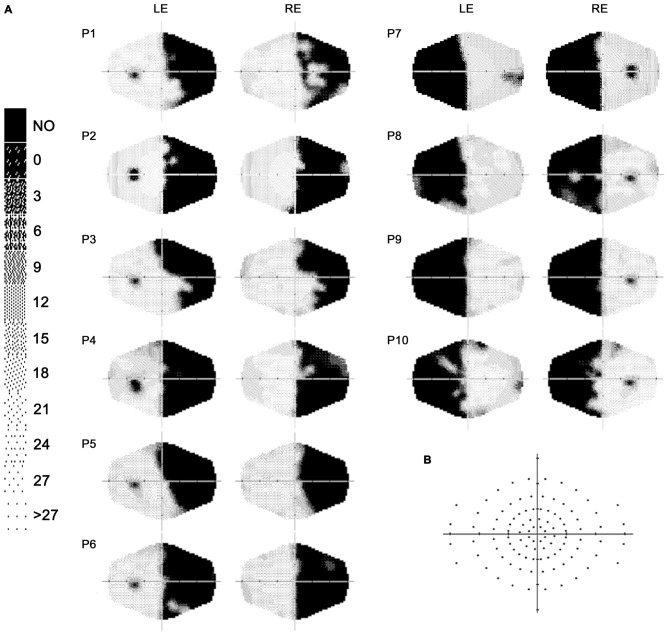
**(A)** Computerized automated visual perimetry (Medmont M700 automated perimetry apparatus, Melbourne, Australia). Axial hash marks denote 10 visual degree increments; color map reports decibel values; LE = left eye, RE = right eye. **(B)** Schematic view of the visual field maps, depicting the locations of visual stimulation.

**Figure 2:**
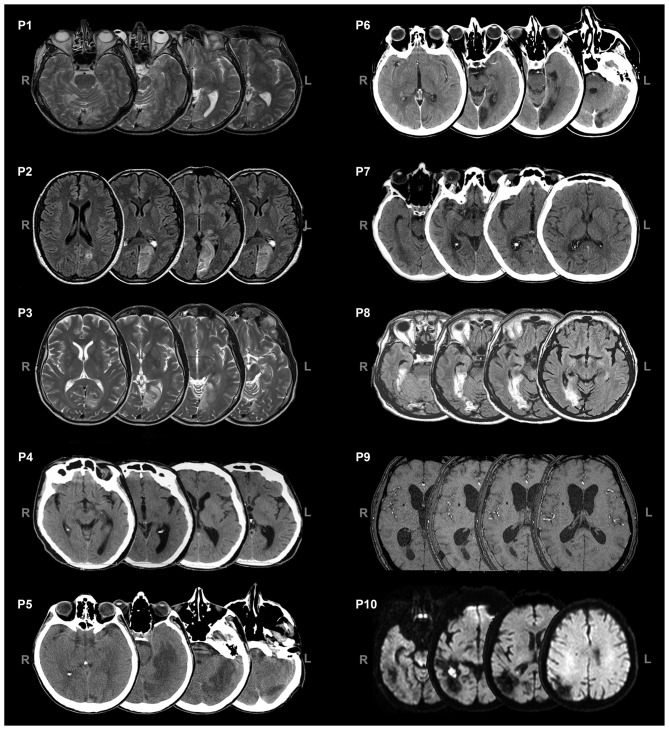
**Axial views of CT/MRI scans of the patients.** L = left, R = right.

Ten patients (2 females, mean age = 49.8 years, SD = 13.7) with chronic visual field defects (mean time since lesion at the first evaluation = 6.4 months; Table 1) took part in the study. Patient were selected based on reported visual field deficits, the availability of a full visual perimetry (Figure 1) and CT/MRI scans of the lesion (Figure 2). Patients with right-lesions were tested using the Behavioral Inattention Test for neglect assessment (Wilson et al., 1987), to ensure performance was in the normal range. All patients showed normal hearing and normal or corrected-to normal-visual acuity. Patients were informed about the procedure and the purpose of the study, and gave written informed consent. The study was designed and performed in accordance with the ethical principles of the Declaration of Helsinki, and was approved by the Ethics Committee of the Psychology Department at the University of Bologna.

**Table 1:** **Demographic and clinical data**.

ID	Sex	Age	Education	Onset	Lesion site	Etiology
P1	M	57	13	7	Left occipital	Ischemic
P2	M	39	13	3	Left occipital	Ischemic
P3	M	44	13	3	Left temporo-occipital	Ischemic
P4	M	33	13	11	Left temporal	Ischemic
P5	M	50	8	6	Left thalamus and temporo-occipital	Ischemic
P6	F	54	18	7	Left temporo-occipital	Ischemic
P7	F	37	13	12	Right temporo-parietal-occipital	Ischemic
P8	M	69	8	3	Right temporo-occipital	Ischemic
P9	M	41	11	9	Right temporo-occipital	AVM
P10	M	74	23	3	Right temporo-parietal-occipital	Hemorrhagic

*M, male; F, female; Age in years; Education in years; Onset of lesion prior to first testing session in months; AVM, arteriovenous malformation*.

### Experimental Design

Patients completed both a clinical assessment and an oculomotor assessment at three time points, i.e., before treatment (B), immediately after treatment (P) and in a follow-up session (F; mean time after training = 8 months, SD = 3.02 months). Notably, the clinical and oculomotor measures used in the present study have been demonstrated to be resistant to practice effects, as shown by patients’ stable performance in test-retest assessments (Bolognini et al., 2005; Passamonti et al., 2009; Dundon et al., 2015). As a consequence, to reduce the testing time and patient fatigue, patients were not tested with a second control baseline in the present study.

Instead, electroencephalography (EEG) measures were collected at four time points: baseline 1, i.e., before treatment (B1), control baseline 2, i.e., 2 weeks after B1 and immediately before treatment (B2), immediately after treatment (P) and in a follow-up session (F; mean time after training = 8 months, SD = 3.02 months). The second baseline (B2) was included to control for any possible effects of merely repeating the test (i.e., practice effects).

#### Clinical Measures

Patients completed a neuropsychological assessment (Bolognini et al., 2005; Passamonti et al., 2009), measuring visual detection, visual scanning, reading abilities and self-perceived disability in daily activities.

*Visual detection—Unisensory visual test*. In a light-attenuated room, patients detected the presence of a light stimulus (red LED; luminance: 90 cd/m^2^; diameter: 0.5 cm) presented on the horizontal meridian of the treatment apparatus (height: 30 cm, length: 200 cm; Figure 4A), by pressing a button. The visual stimulus could appear at one of eight eccentricities (56°, 40°, 24° and 8° bilaterally). Patients were asked to keep their head fixed, oriented towards the center of the apparatus. However, they were free to move their eyes. An experimenter monitored when eyes were centered and administered the light stimulus (100 ms). Patients performed three blocks of 120 trials (12 trials at each eccentricity and 24 catch trials, i.e., no light stimulus). The accuracy (i.e., the percentage of correctly detected targets) at each eccentricity constituted the outcome metric.

**Figure 3:**
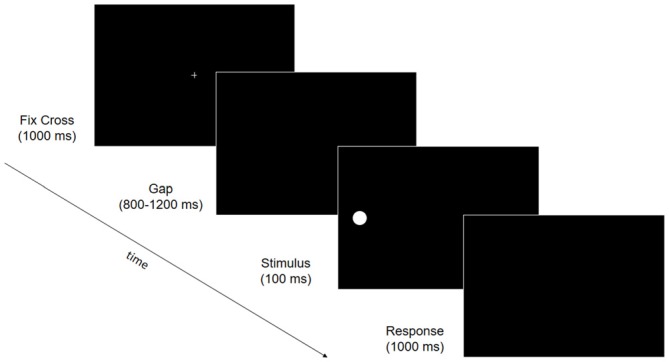
**Trial structure of the electroencephalography (EEG) behavioral task.** Fixation cross (1000 ms) was followed by a gap ranging from 800 to 1200 ms. A stimulus was then presented for 100 ms at one of six possible locations (upper, median or lower, 15° to the right or left visual field) followed by a response window of 1000 ms in which participants were asked to press space-bar when they detected the visual stimulus.

**Figure 4:**
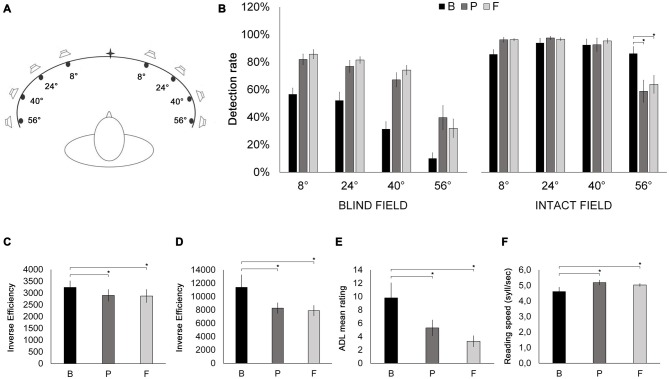
**(A)** Schematic bird’s eye representation of the apparatus used for the Visual detection—Unisensory visual test and the audio-visual training. Patients were placed at the center of a concave ellipse (200 cm in width and 30 cm in height) in which eight LED lights and eight piezoelectric loudspeakers were positioned at increasing eccentricities (8°, 24°, 40° and 56° to the left and to the right) with respect to the center. During the Visual detection—Unisensory visual test, only LED stimuli were used. **(B)** Results of the Visual detection—Unisensory visual test. Detection rates (% correct stimulus detections) are depicted as a function of stimulus eccentricity (8°, 24°, 40° and 56°) and visual field (blind field, intact field), at B (black bars), P (dark gray bars) and F (light gray bars) sessions. **(C)** Visual search. Inverse efficiency scores (reaction time/accuracy) for the E-F test as a function of testing session (B, P, F). **(D)** Visual search. Inverse efficiency scores (reaction time/accuracy) for the Triangles test as a function of testing session (B, P, F). **(E)** Mean ratings from the Activity of Daily Living inventory as a function of testing session (B, P, F). **(F)** Reading text task. Reading speed (syllables/second) as a function of testing session (B, P, F). Error bars report standard error of the mean. Asterisks indicate significant comparisons (*p* < 0.05).

*Visual search—E-F test* (modified from Zihl, 2000; Bolognini et al., 2005). A personal computer running a custom Software (C.I.R.O.) developed in C++, using QT libraries^1^, was used to present stimuli and record responses. One target stimulus (green capital F; 2° × 2°; RGB values: 0, 163, 0; luminance: 15 cd/m^2^) and 20 distractors (green capital E; 2° × 2°; RGB values: 0, 163, 0; luminance: 15 cd/m^2^) were displayed on a projector screen (NEC V260X projector) randomly within a 52° × 45° array on a black background (RGB values: 0, 0, 0; luminance: 0.5 cd/m^2^). Patients (at a distance of 120 cm from the projector screen) responded as quickly as possible if the target was present or not, with one of two buttons on the mouse. Patients performed one block of 20 trials—16 target-present trials and 4 target-absent trials (i.e., catch trials). Accuracy and response times were recorded, and inverse efficiency scores (IES = response time divided by the percentage of accurate detections) were computed.

*Visual search—Triangles test* (modified from Zihl, 2000; Bolognini et al., 2005). Using the same procedure as above, patients were asked to count targets (yellow triangles; 2° × 2°; RGB values: 253, 253, 110; luminance: 31 cd/m^2^), amongst distractors (yellow squares; 2° × 2°; RGB values: 253, 253, 110; luminance: 31 cd/m^2^) displayed against a black background (RGB values: 0, 0, 0; luminance: 0.5 cd/m^2^). Patients pressed a button when they were able to indicate the number of targets in the array, which marked the response time. They then verbally declared their response, which was noted by the experimenter on a response sheet. IES (IES = response time divided by the percentage of accurate detections) were computed.

*Reading text task* (Bolognini et al., 2005). The text was a short story in Italian (330 syllables), presented on a computer monitor (visual scene: 30° × 24°). Four different stories could be presented and were counterbalanced between subjects and testing sessions. The graphical and lexical characteristics (6–8 lines for each paragraph; distance between lines: 1.5 cm; 5–6 words per line; font: Arial 40) of the chosen texts were equivalent. Subjects were asked to read aloud, and reading time was measured (syllables/s).

*Self-report—Activities of Daily Living Inventory* (ADL; modified from Kerkhoff et al., 1992; Bolognini et al., 2005). Patients were asked to complete a 10-item, 5-point Likert scale questionnaire exploring the dimensions of visual impairment in daily life. Raw mean scores constituted the outcome metric.

#### Oculomotor Measures

Eye movements were assessed while patients performed the *Visual search—Number test* (modified from Bolognini et al., 2005). Eight stimulus arrays were presented, depicting the numbers 1–15 (2° × 2°; printed in red, RGB values: 251, 0, 55; luminance: 11 cd/m^2^) on a black background (RGB values: 0, 0, 0; luminance: 0.5 cd/m^2^), in random positions. Patients identified each number in ascending order while eye movements were recorded.

Eye movements were recorded using a Pan/Tilt optic eye-tracker (Eye-Track ASL-6000) which registers real-time gaze at 60 Hz. The recording was performed in a dimly lit room. The patient’s dominant eye was illuminated with invisible infrared light, and the reflections were recorded by a video camera positioned 60 cm from the eye. The experimenter monitored online the position of patient’s eye in the visual scene, during the task. Before collecting data from each patient, the equipment was calibrated using a 9-point grid. Patients were asked to fixate successively on each of a series of small dots arranged on three lines. Fixation time at each dot position was at least 3 s.

Data from eye movement recordings were quantitatively analyzed with respect to the number of fixations and saccadic speed (saccadic amplitude/saccadic duration). In addition, mean exploration time was taken as a behavioral measure of visual exploration.

#### EEG Measures

EEG data were recorded at B1, B2, P and F while patients performed a simple visual detection task. During the task, patients were placed 57 cm away from a 17” PC monitor (refresh rate: 60 Hz). Stimuli were presented on a PC running Presentation Software (Version 0.60)^2^. A target stimulus (white, RGB values: 255, 255, 255; luminance: 129 cd/m^2^; 1° diameter circle) appeared against a black background (RGB values: 0, 0, 0; luminance: 0.5 cd/m^2^) at one of six locations: 15° right or left of the central fixation cross, and on the midline (i.e., horizontally aligned with the central fixation cross), or in the upper or lower quadrant (i.e., 13° above or below the midline). Each trial consisted of a central fixation cross (1000 ms), followed by a gap (800–1200 ms), a target (100 ms) and a response window (1000 ms, Figure 3). Catch trials (i.e., a fixation cross followed by a gap, but no stimulus) were included, to control for false positives. Patients could not move their eyes and were instructed to maintain central eye-fixation throughout the entire trial, and to detect the presence of the stimulus, pressing a response button as quickly as possible. Patients performed 27 blocks of 30 trials (an average of 115 trials at each visual location, and 115 catch trials). EEG data were recorded with Ag/AgCl electrodes (Fast’n Easy-Electrodes, Easycap, Herrsching, Germany) from 27 electrode sites (Fp1, F3, F7, FC1, FC5, C3, T7, CP1, CP5, P3, P7, O1, Fz, Cz, Pz, Fp2, F4, F8, FC2, FC6, C4, T8, CP2, CP6, P4, P8, O2) and the right mastoid. The left mastoid was used as reference, while the ground electrode was positioned on the right cheek. Vertical and horizontal electrooculogram (EOG) components were recorded from above and below the left eye, and from the outer canthus of both eyes. Data were recorded with a band-pass filter of 0.01–100 Hz and amplified by a BrainAmp DC amplifier (Brain Products, Gilching, Germany). The amplified signals were digitized at a sampling rate of 500 Hz, offline filtered with a 40 Hz low-pass filter, and then analyzed using custom routines in Matlab 7.12.0.635 (R2011a; The Mathworks, Natick, MA, USA) and EEGLAB v10.2.5.8b (Delorme and Makeig, 2004). Data from all electrodes were re-referenced offline to the average of both mastoids. Stimulus triggers were located within the continuous EEG waveform and used to anchor the epochs (−200 ms to 900 ms; baseline window −100 ms to 0 ms pre-stimulus). Epochs containing artifacts were excluded using methods from the EEGLAB toolbox (Delorme et al., 2007). Epochs with large EEG peaks (greater than an individually adjusted threshold, mean 242 μV) and with improbable data (joint probability of a trial >5 × SD) were also excluded (mean: 41.9 epochs per participant per session). Remaining vertical EOG artifacts were corrected using a regression approach (Gratton et al., 1983). Finally, epochs were discarded if horizontal saccadic movements (>30 μV on horizontal EOG channels) were registered 0 ms to 200 ms post-stimulus onset, to control for eye-movements explaining stimulus detection (mean: 55.8 epochs per participant per session). In total, 12% of epochs were excluded; remaining epochs were averaged.

The P3 component was quantified as the mean amplitude in a time window between 370 and 410 ms post stimulus presentation.

Epochs were averaged for the entire group: electrodes were swapped cross-hemispherically for patients with lesions to the left hemisphere. Thus, the data were analyzed as if all participants were right-lesioned. Scalp topography at B1 in the chosen time window showed a maximal positive inflection over electrodes CP1, P3 and Pz (Figure 6A). Data from these electrodes were therefore used for statistical analysis.

**Figure 5:**
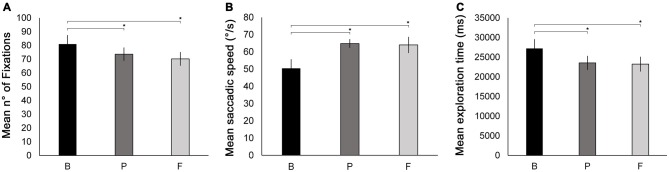
**Oculomotor measures recorded during the Number visual search test.** Mean number of fixations **(A)**, mean saccade speed **(B)** and mean exploration time **(C)** are reported as a function of testing session (B, P, F). Error bars report standard error of the mean. Asterisks indicate significant comparisons (*p* < 0.05).

**Figure 6:**
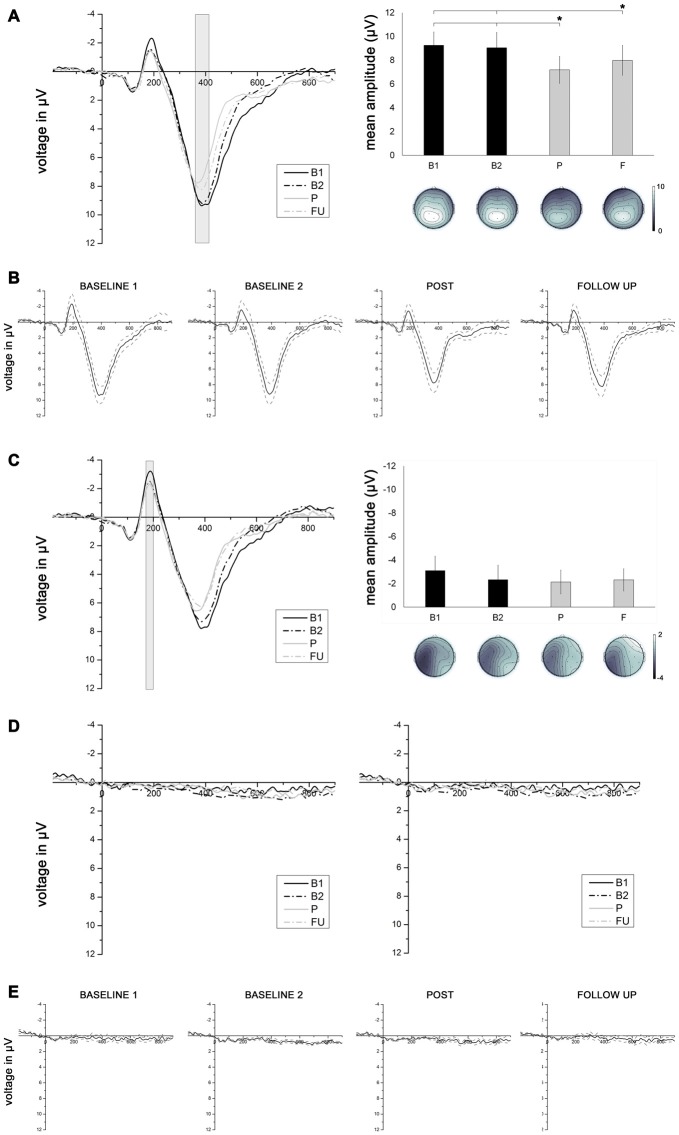
**(A)** Left panel depicts grand average event-related potentials (ERPs) averaged across electrodes Pz, P3 and CP1, elicited by stimuli presented in the intact visual field, as a function of session (B1, B2, P, F). Right panel depicts mean P3 amplitudes (with corresponding topographies) measured in a time window between 370 and 410 ms as a function of testing session (B1, B2. P, F). Asterisks connected with lines indicate significant comparisons (*p* < 0.05). **(B)** Grand average ERPs averaged across electrodes Pz, P3 and CP1 (solid line), elicited by stimuli presented in the intact visual field, for each of the four testing sessions, with corresponding standard errors (dotted lines). **(C)** Left panel depicts grand average ERPs averaged across electrodes C3, CP5, P7 and P3, elicited by stimuli presented in the intact visual field, as a function of session (B1, B2, P, F). Right panel depicts mean N1 amplitudes (with corresponding topographies) measured in a time window between 180 and 200 ms as a function of testing session (B1, B2, P, F). **(D)** Grand average ERPs elicited by stimuli presented to the blind visual field averaged across electrodes Pz, P3 and CP1 (left panel) and across electrodes C3, CP5, P7 and P3 (right panel), as a function of testing session (B1, B2, P, F). **(E)** Grand average ERPs averaged across electrodes Pz, P3 and CP1 (solid line), elicited by stimuli presented in the blind visual field, for each of the four testing session, with corresponding standard errors (dotted lines).

Given that early sensory components such as the visual N1 can be modulated by visual spatial attention (for a review, see Hillyard et al., 1998), we also analyzed this component. The N1 was quantified as the mean amplitude in a time window between 180 and 200 ms. Scalp topography at B1 in the chosen time window showed a maximum negative inflection (Figure 6C) over electrodes C3, CP5, P7 and P3; data from these electrodes were used for the statistical analysis.

#### Training

The training lasted 10 days (4 h of training per day). Patients were presented with three different kinds of sensory stimulation (Figure 4A): (i) unisensory visual (UV; 100 ms red LED light; luminance: 90 cd/m^2^; diameter: 0.5 cm); (ii) unisensory auditory (UA; 100 ms, 80 dB white noise); and (iii) multisensory audio-visual (MAV; UV and UA simultaneously at the same location). Patients were asked to fixate centrally and performed visual explorations, while the head remained stationary. When any visual stimulus (UV or MAV) was observed, patients were asked to respond with a button-press. Stimuli were disproportionately allocated to the hemianopic side, to encourage exploration of this field (for further details on the training protocol, please see: Bolognini et al., 2005; Passamonti et al., 2009; Dundon et al., 2015; for the apparatus, see also the “Visual detection—Unisensory visual test” paragraph under the “Materials and Methods” Section). Patients performed approximately 30 blocks per day, of 48 trials each (12 UV; 12 UA and 24 MAV).

## Results

The effects of treatment were tested with repeated measures ANOVAs on clinical, oculomotor and electrophysiological measures. To compensate for violations of sphericity, Greenhouse-Geisser corrections (Greenhouse and Geisser, 1959) were applied whenever appropriate; corrected *p*-values (but uncorrected degrees of freedom) are reported. Partial eta-squared ($\eta_{p}^{2}$) effect sizes are also reported. *Post hoc* comparisons were conducted using the Newman-Keuls test.

### Clinical Measures

*Visual detection—Unisensory visual test*. Raw accuracy scores were analyzed with a 2 × 3 × 4 ANOVA, with visual field (hemianopic, intact), session (B, P, F) and location (56°, 40°, 24°, 8°) as within-subjects factors. The main effects of visual field (*F*_(19)_ = 51.85, *p* = 0.00005, $\eta_{p}^{2}$ = 0.852), session (*F*_(2,18)_ = 30.31; *p* = 0.000003, $\eta_{p}^{2}$ = 0.771) and location (*F*_(3,27)_ = 127.83, *p* = 0.0000000004, $\eta_{p}^{2}$ = 0.934) were significant. Notably, the three-way interaction between visual field, session and location was also significant (*F*_(6, 54)_ = 3.21; *p* = 0.048, $\eta_{p}^{2}$ = 0.262). Thus, two separate 3 × 4 ANOVAs were conducted, for the hemianopic and intact visual fields, respectively, with the factors session (B, P, F) and location (56°, 40°, 24°, 8°). The ANOVA on the hemianopic field revealed a significant effect of session (*F*_(2,18)_ = 36.52; *p* = 0.000001, $\eta_{p}^{2}$ = 0.804): accuracy scores significantly increased from B (37.6%) to P (66.4%; *p* = 0.0002) and from B to F (68.3%; *p* = 0.0001). No significant difference was instead observed between P and F (*p* = 0.637). Also, the main effect of location was significant (*F*_(3,27)_ = 49.61; *p* = 0.000002, $\eta_{p}^{2}$ = 0.846): accuracy was significantly lower at 56° (27.2%), compared to 40° (57.6%; *p* = 0.0001), 24° (70.2%; *p* = 0.0001) and 8° (74.8%; *p* = 0.0002), and also lower at 40° compared to 24° (*p* = 0.007) and 8° (*p* = 0.001). The session × location interaction was not significant (*F*_(6,54)_ = 1.82; *p* = 0.156, $\eta_{p}^{2}$ = 0.163). The ANOVA on the intact field revealed a significant interaction between session and location (*F*_(6, 54)_ = 6.65; *p* = 0.004, $\eta_{p}^{2}$ = 0.426). *Post hoc* comparisons revealed that at 56°, compared to B (86.2%), accuracy was significantly reduced at P (58.8%, *p* = 0.0001) and at F (63.8%; *p* = 0.0002), while no significant difference was found between P and F (*p* = 0.32). At the remaining three stimulus locations, accuracy was unchanged across all three testing sessions (all *p*-values > 0.352, Figure 4B). An ANOVA with the factor session (B, P, F) comparing the percentages of false alarms revealed no significant differences between sessions (*F*_(2,18)_ = 1.38; *p* = 0.272, $\eta_{p}^{2}$ = 0.166; B: 0%; P: 2%; F: 1%).

*Visual search—E-F test*. The ANOVA on IE scores with the factor session (B, P, F) revealed a significant main effect of session (*F*_(2,18)_ = 4.47, *p* = 0.042, $\eta_{p}^{2}$ = 0.332), compared to B (3242 ms), IE scores at P (2902 ms) and at F (2875 ms) were significantly lower (*p* = 0.023 and *p* = 0.039, respectively; Figure 4C), reflecting a post-treatment improvement in scanning efficiency, with no difference between P and F (*p* = 0.844). The ANOVA with the factor session (B, P, F) on the percentage of false alarms revealed no significant effect of session (*F*_(2,18)_ = 0.995, *p* = 0.344, $\eta_{p}^{2}$ = 0.117; B: 0%; P: 5%; F: 0%).

*Visual search—Triangles test*. The ANOVA on IE scores with the factor session (B, P, F) revealed a significant main effect of session (*F*_(2,18)_ = 7.29, *p* = 0.022, $\eta_{p}^{2}$ = 0.447), compared to B (11,390 ms), IE scores at P (8274 ms) and at F (7894 ms) were significantly lower (*p* = 0.006 and *p* = 0.007, respectively; Figure 4D), reflecting more efficient visual scanning at post-treatment and follow-up sessions. No difference was observed between P and F (*p* = 0.709). The ANOVA with the factor session (B, P, F) computed on the percentage of false alarms revealed no significant effect of session (*F*_(2,18)_ = 0.00, *p* = 1.00, $\eta_{p}^{2}$ = 0.000; B: 0%; P: 0%; F: 0%).

*ADL—*The ANOVA on ADL scores with the factor session (B, P, F) revealed a significant main effect of session (*F*_(2,18)_ = 13.21, *p* = 0.003, $\eta_{p}^{2}$ = 0.595). ADL scores were significantly lower at P (5.3) and at F (3.3), compared to B (9.8; *p* = 0.003 and *p* = 0.0004, respectively), showing a significant improvement in the quality of patients’ daily living, both immediately after training and at the follow-up session. In contrast, ADL scores were not significantly different between P and F (*p* = 0.14; Figure 4E).

*Reading text task—*An ANOVA on reading speed with the factor session (B, P, F) revealed a significant main effect of session (*F*_(2,18)_ = 4.68, *p* = 0.047, $\eta_{p}^{2}$ = 0.341), showing significantly improved reading speed at P (5.19 syllables/s) and at F (5.03 syllables/s), compared to B (4.61 syllables/s; *p* = 0.02 and *p* = 0.04, respectively; Figure 4F), while no difference was found between P and F (*p* = 0.44).

### Oculomotor Measures

ANOVAs with the factor session (B, P, F) were conducted separately for each oculomotor parameter measured (see “Oculomotor Measures” under “Materials and Methods” Section). The ANOVA on the number of fixations revealed a significant main effect of session (*F*_(2,18)_ = 5.23, *p* = 0.038, $\eta_{p}^{2}$ = 0.367). The number of fixations was significantly reduced at P (73.6) and at F (70.2) compared to B (80.9, *p* = 0.044 and *p* = 0.014, respectively). No significant difference was found between P and F (*p* = 0.329, Figure 5A). Also, the ANOVA on mean saccadic speed revealed a significant main effect of session (*F*_(2,18)_ = 6.22, *p* = 0.013, $\eta_{p}^{2}$ = 0.408). Saccades were significantly faster at P (64.81°/s) and at F (64.00°/s) compared to B (50.45°/s, *p* = 0.015 and *p* = 0.008 respectively; Figure 5B). No significant difference was found between P and F (*p* = 0.862).

In addition, the ANOVA conducted on mean exploration times revealed a significant main effect of session (*F*_(2,18)_ = 9.19, *p* = 0.007, $\eta_{p}^{2}$ = 0.50). Mean exploration time was significantly lower at P (23.5 s) and at F (23.2 s) compared to B (27.2 s; *p* = 0.002 and *p* = 0.003, respectively), while no difference was observed between P and F (*p* = 0.766). This indicates a significant post-treatment improvement in visual exploration that was maintained at the follow-up session (Figure 5C).

### EEG Measures

*Behavioral Data*. Since during the task patients were asked to fixate centrally and not to move their eyes, they detected, as expected, a low number of stimuli in the hemianopic field (6% at B1, 6% at B2, 7% at P and 7% at F). Analyses on accuracy, response times and detection sensitivity were therefore performed only for stimuli presented in the intact visual field, using 4 × 3 ANOVAs with session (B1, B2, P, F) and location (upper, middle, lower) as factors. Neither accuracy (*F*_(3,27)_ = 1.24, *p* = 0.304, $\eta_{p}^{2}$ = 0.121; B1 = 98%, B2 = 97%, *P* = 98%, *F* = 98%), response time (*F*_(3,27)_ = 1.02, *p* = 0.375, $\eta_{p}^{2}$ = 0.102; B1 = 418.5 ms, B2 = 422.9 ms, *P* = 408.7 ms, *F* = 406.3 ms) nor detection sensitivity (*F*_(3,27)_ = 0.94, *p* = 0.417, $\eta_{p}^{2}$ = 0.094; B1 = 4.75, B2 = 4.78, *P* = 4.75, *F* = 4.55) changed across sessions, nor were there any significant interactions involving session and location (all *p*-values > 0.105).

*EEG Data*. No worthwhile ERPs were elicited by stimuli in the hemianopic field Figures 6D,E). As a consequence, only ERPs elicited by visual stimuli presented in the intact field were analyzed. A 4 × 3 × 3 ANOVA with the factors session (B1, B2, P, F), electrode (Pz, P3, CP1) and location (upper, middle, lower) compared the effect of treatment on the P3 component elicited by stimuli presented in the intact visual field. The main effect of session was significant (*F*_(3,27)_ = 7.61, *p* = 0.0008, $\eta_{p}^{2}$ = 0.458). The mean P3 amplitude at session P (7.19 μV) was significantly lower compared to the mean P3 amplitude at B1 (9.25 μV; *p* = 0.002) and at B2 (9.05 μV; *p* = 0.002). The mean P3 amplitude at session F (7.99 μV) was also significantly lower than the mean P3 amplitudes at B1 (*p* = 0.04) and B2 (*p* = 0.04). There was no significant difference in P3 amplitude between B1 and B2 (*p* = 0.689), or between P and F (*p* = 0.117, Figures 6A,B).

To control for other possible effects of the training on early sensory components that are known to be modulated by visuo-spatial attention (i.e., the N1 component; for a review, see Hillyard et al., 1998), a 4 × 4 × 3 ANOVA with the factors session (B1, B2, P, F), electrode (C3, CP5, P7, P3) and location (upper, middle, lower) was conducted on the N1 component elicited by stimuli presented in the intact visual field. The results revealed no main effect of session (*F*_(3,27)_ = 1.36, *p* = 0.283, $\eta_{p}^{2}$ = 0.131), suggesting that the mean N1 amplitude remained constant over the four testing sessions (B1 = −3.12 μV; B2 = −2.34 μV; *P* = −2.14 μV; *F* = −2.31 μV; see Figure 6C).

## Discussion

In everyday life, hemianopic patients continuously experience asymmetric visual inputs, which could lead to an imbalance of attentional resource allocation towards the intact visual field (Tant et al., 2002). Multisensory audio-visual stimulation can reduce this attentional imbalance and improve clinical signs of hemianopia. Indeed, the present results confirm previous findings (Bolognini et al., 2005; Passamonti et al., 2009; Dundon et al., 2015) and provide new evidence for the long-term efficacy of the audio-visual training in both ameliorating visual performance and reducing the attentional bias towards the ipsilesional visual field. At the behavioral level, we observed an improvement in visual search abilities, an increase in visual detection in the hemianopic field and improvements in self-perceived disability in daily life activities, at both the P and F sessions. Furthermore, oculomotor parameters during visual search revealed a reduction in the number of fixations, an increase in mean saccadic speed and a reduction in the mean exploration time at P and F sessions, suggesting the implementation of more organized visual exploration strategies. At the electrophysiological level, we found a reduction in the posterior-parietal P3 component elicited by simple visual detection in the periphery of the intact visual field, both at the P session and at the F session. In addition, no differences were found between B1 and B2 sessions, or between P and F sessions, dismissing any possible explanation of the results as practice effects, and confirming the long-term duration of the modifications induced by treatment.

The observed improvements in clinical and oculomotor parameters seem to rely on the spared retino-collicolo-dorsal pathway, which is known to play a critical role in integrating audio-visual stimuli (Stein and Meredith, 1993; Calvert, 2001; Meienbrock et al., 2007; Bertini et al., 2008, 2010; Leo et al., 2008a; Maravita et al., 2008; Nardo et al., 2014). The relevance of the SC in mediating the post-training ameliorations is also suggested by the observation that improvements are seen when orienting responses towards the blind field are possible. Indeed, the SC is relevant in target selection and in the initiation and execution of saccades (Krauzlis et al., 2004), and contributes to oculomotor planning (Arikuni et al., 1980; Barbas and Mesulam, 1981). In contrast, when fixation is required and eye movements are not allowed, the activity of the caudal SC is suppressed and the saccadic generation is prevented (Munoz and Guitton, 1989, 1991; Munoz and Wurtz, 1993a,b; Munoz and Istvan, 1998; for a review, see Gandhi and Katnani, 2011). Moreover, electrophysiological findings corroborate the hypothesis of the pivotal role of the retino-collicolo-dorsal pathway as the neural substrate for post-training improvements, showing that hemianopic cats, after similar audio-visual training, can recover visual orienting and visual responsiveness in the SC neurons. In addition, repeated exposure to audio-visual pairs has been shown to increase multisensory responses in the SC (Yu et al., 2009, 2012, 2016). Interestingly, systematic multisensory stimulation can also uncover the responsiveness of the SC neurons to stimuli in the unisensory visual modality (Yu et al., 2009, 2012), showing that audio-visual stimulation can be effective at inducing plastic changes in the responses of SC neurons.

In addition, the neural network involving the SC, extrastriate and dorsal-parietal cortices is known to have a crucial role not only in orienting movements of the eyes and the head towards visual stimuli, but also in controlling visual spatial attention (Krauzlis et al., 2013). This seems in line with the present finding of a reduction in the amplitude of the P3 component in response to stimuli presented in the intact field, which seems to reflect a reallocation of spatial attentional resources after audio-visual training. Indeed, although no consensus has been reached on the exact processes underlying the P3 (Kok, 2001), this component has been interpreted as an index of attentional resource allocation (Isreal et al., 1980; Wickens et al., 1983). Specifically, the P3 has been reported to involve endogenous attention, within a late stage of cortical visual processing (Hopfinger and West, 2006). Moreover, attentional orienting has been consistently shown to influence P3 amplitude (for a review, see Polich, 2007).

Alternatively, the observed decrease in the P3 amplitude could be due to a reduced recruitment of active neurons, reflecting a facilitatory effect of the training on the execution of the task. However, the hypothesis of a simple post-training facilitatory effect seems unlikely, since, after training, also attentional costs can be observed. Indeed, the observed reduction of the P3 amplitude was associated with a post-treatment reduction in detection accuracy at the most peripheral eccentricity (56°) of the intact hemifield in the unisensory visual test. This corroborates the hypothesis of a reduction in attentional resource allocation toward the intact field after training. Indeed, the implementation of a more efficient oculomotor strategy after training might have increased compensatory saccadic planning towards the hemianopic field, inducing a consequent shift of attention from the intact to the blind field. This seems in line with evidence suggesting that preparation of saccades evokes visual attentional shifts towards the targeted location of the saccades (for a review, see Zhao et al., 2012).

The stability at the follow-up session of the post-training improvements at the clinical and the oculomotor levels, as well as the electrophysiological changes, is extremely relevant to the neural plasticity of the visual system. Indeed, these findings reveal that systematic audio-visual stimulation with hemianopic patients can induce a long-term implementation of efficient compensatory oculomotor strategies and a long-lasting reallocation of attentional resources, therefore suggesting a stable plastic change of the neural circuit (i.e., the retino-colliculo-dorsal pathway) subserving these effects. This seems in line with recent electrophysiological findings showing that when the neurons of the SC, deprived of any early sensory experience, are repeatedly exposed to spatially coincident audio-visual stimuli, they acquire stable multisensory integrative responses, which are maintained without further multisensory experience for more than 1 year (Xu et al., 2012).

Overall, these results show that systematic audio-visual multisensory stimulation can promote long-term plastic changes in hemianopic patients, with stable and long-lasting beneficial effects resulting in ameliorations in their quality of life.

## Author Contributions

PAG collected and analyzed data; PAG, EL and CB designed the experiment and wrote the article.

## Conflict of Interest Statement

The authors declare that the research was conducted in the absence of any commercial or financial relationships that could be construed as a potential conflict of interest.

## Footnotes

^1^http://www.qt.io/

^2^www.neurobs.com
